# A fixation method for the optimisation of western blotting

**DOI:** 10.1038/s41598-019-43039-3

**Published:** 2019-04-30

**Authors:** Jing Xu, Hebin Sun, Guoling Huang, Gang Liu, Zhi Li, Hui Yang, Lingling Jin, Xiaolin Cui, Lei Shi, Tonghui Ma, Akihiko Kameyama, Weijie Dong

**Affiliations:** 1 College of Basic Medical Sciences, Dalian Medical University, Dalian, 116044 Liaoning China; 2 Clinical Laboratory, Dalian Municipal Central Hospital, 826-Xinan Road, Shahekou District, Dalian city, Liaoning 116033 China; 3 Biotechnology Research Institute for Drug Discovery, National Institute of Advanced Industrial Science and Technology (AIST), Open Space Laboratory C-2, 1-1-1 Umezono, Tsukuba, Ibaraki 305-8568 Japan

**Keywords:** Proteomics, Cancer

## Abstract

Western blotting is the most extensively used technique for the identification and characterisation of proteins and their expression levels. One of the major issues with this technique is the loss of proteins from the blotted membrane during the incubation and washing steps, which affects its sensitivity and reproducibility. Here, we have optimised the fixation conditions for immunoblotting and lectin blotting on electroblotted polyvinylidene difluoride and nitrocellulose membranes, using a combination of organic solvents and heating. Loss of proteins from polyvinylidene difluoride membranes was greatly reduced using this approach, the intensity of lectin blotting and immunoblotting was shown to increase 2.8- to 15-fold and 1.8- to 16-fold, respectively, compared with that samples without treated. Using the optimised method, cystic fibrosis transmembrane regulator and hypoxia-inducible factor 1, two difficult-to-analyse proteins with important physiological and pathological roles, were effectively detected. Additionally, it may help the identification of novel diagnostic markers for prostate cancer.

## Introduction

Cellular proteome is involved in the vast majority of biological functions^1^, and proteins play an integral role as the direct executors and regulators of these functions^2^. In eukaryotes, approximately half of all proteins are glycosylated^3^. Alterations in the expression and glycosylation of serum proteins can provide information regarding disease stage and malignancy. Many routine clinical tests utilise the levels of circulating proteins and glycoproteins for diagnostic or prognostic purposes, including the analyses of alanine aminotransferase (ALT) and aspartate aminotransferase (AST) in liver function^4^, and the pancreatic cancer biomarkers, carcinoembryonic antigen (CEA) and CA 19-9^5^. Therefore, the characterisation of proteins, including glycan profiling, is crucial for biomarker research, and may provide valuable insights into the disease mechanisms and pathogenesis.

Western blotting is the most widely used method for specific protein detection. Native or denatured proteins are first separated using gel electrophoresis and then transferred to a support material, such as polyvinylidene difluoride (PVDF) or nitrocellulose membranes, for immunostaining (immunoblotting) or lectin staining (lectin blotting: LB). Immunoblotting is widely used as a convenient detection method, for example, for the detection and semi-quantitation of an antigen, and the determination of the relative molecular weight of a polypeptide chain and the extraction efficiency of a target antigen^6^. LB is a useful biochemical technique for the detection of characteristic glycosylation patterns, based on the lectin-glycan interactions^7^. However, the sensitivity of detection can be affected by the transfer efficiency and loss of proteins from the membrane during washing. Buffers that contain detergents, such as Tween 20, lead to the removal of hydrophilic proteins from the membranes^8,9^. These issues are common and can lead to a decrease in the detection accuracy and reproducibility. Despite the improvements in the fixative reagents, electrode materials, transfer methods, and other steps in the western blotting process^8,10,11^, several challenges remain.

To avoid protein detachment from electroblotted membrane during subsequent incubations and retain more proteins, improvements on electroblotted membranes and electrophoresis method have been attempted. One fixation method which used 0.5% (v/v) glutaraldehyde for the detection of low molecular-weight acidic and basic isoelectric point (pI) proteins increased the sensitivity of western blotting from 1.5- to 12-fold^12^. Additionally, a new electrophoresis method, supported molecular matrix electrophoresis (SMME), has been developed using PVDF membranes, which does not require any transfer, but uses the fixation step for immunoblotting, applying acetone immersion and/or heating of the membrane after electrophoresis^13,14^. Organic solvents such as acetone and methanol are often used as fixatives for the immunostaining of tissues or cells^15^. Furthermore, for the on-membrane detection, heating of the membranes was reported as a fixation method as well^16^.

Here, we aimed to optimise a fixation method, by immersing the PVDF and nitrocellulose membranes into organic solvents, which was followed by heating, to maximize the amount of proteins retained on electroblotted membranes, thus facilitate the detection of immunoblotting and LB. Furthermore, we used this approach to analyse cystic fibrosis transmembrane regulator (CFTR) expression in human colon carcinoma HT-29 cells and hypoxia-inducible factor 1 (HIF-1) expression in HEK-293T cells, GAPDH in liver tissue of mouse, as well as the serum levels of alpha fetoprotein (AFP) in hepatocellular carcinoma (HCC) patients and the glycosylation status using prostate cancer patient samples.

## Results

### Fixation Prior to Immunostaining

**Figure 1:**
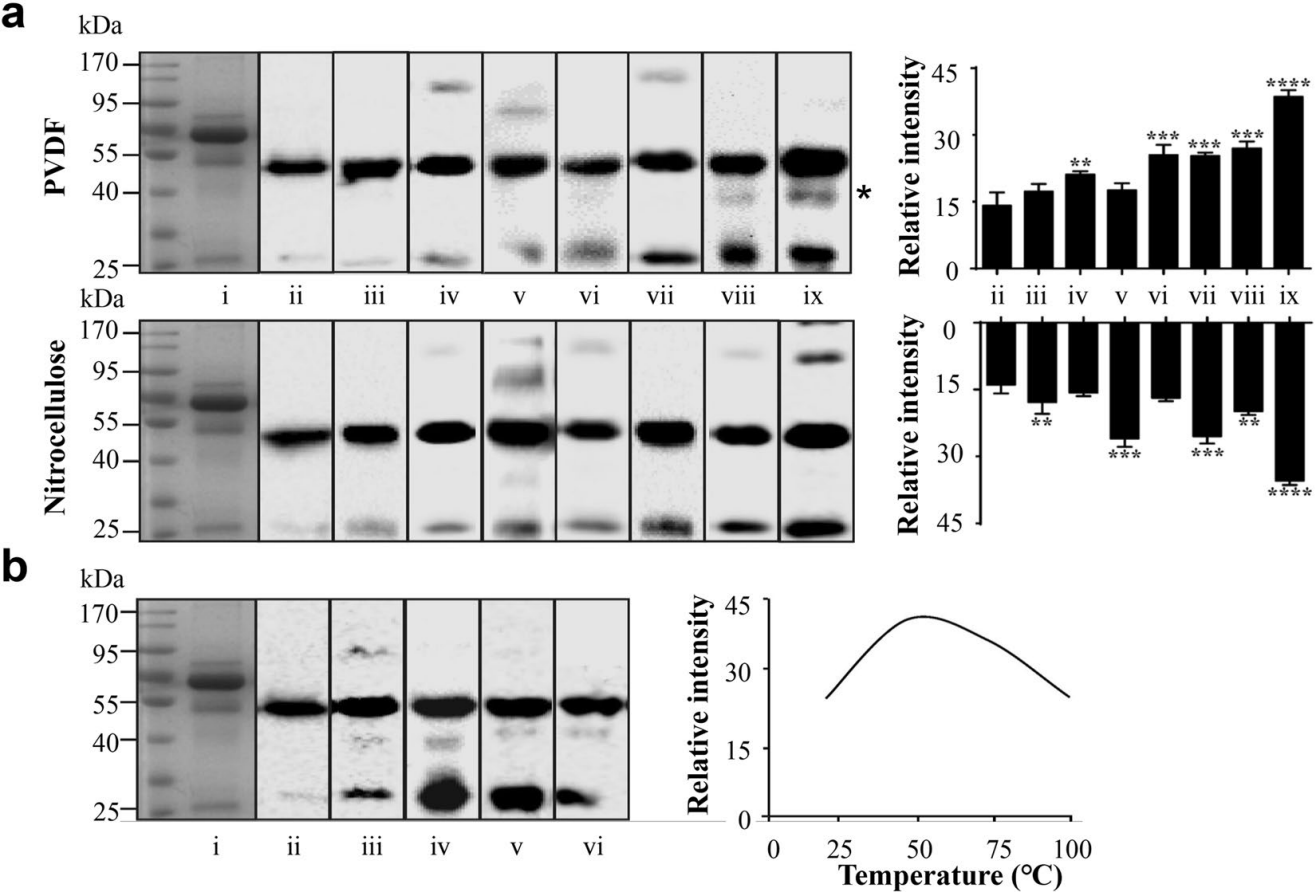
Fixation method-dependent differences in the immunostaining intensity. (**a**) Pooled human serum samples (10 μg) were analysed by 10% SDS-PAGE, and the separated proteins were transferred onto PVDF (top) and nitrocellulose (bottom) membranes, the whole membrane was cutted into eight pieces for subsequently fixation treatments. Representative images, showing anti-human IgG antibody staining after the following treatments: lane i, Coomassie Brilliant Blue (CBB) staining; lane ii, no fixation; lane iii, drying at room temperature; lane iv, heating at 50 °C; lane v, heating at 100 °C; lane vi, immersion into the organic solvents (acetone and 50% methanol for PVDF and nitrocellulose membranes, respectively) at room temperature; lane vii, immersion into the organic solvents at 0 °C; lane viii, immersion into the organic solvents at 0 °C followed by sample heating at 100 °C; lane ix, immersion into the organic solvents at 0 °C followed by sample heating at 50 °C. All treatments were performed for 30 min. The relative intensity was shown on the right (n = 3 individual experiments). (**b**) Proteins were separated by 10% SDS-PAGE, and transferred onto PVDF membranes, the whole membrane was cutted into five pieces for subsequently fixation treatments. Fixed in acetone at 0 °C, and heated at different temperatures for 30 min prior to the immunostaining. Lane i, CBB staining; lane ii, no fixation; lane iii, heating at 25 °C; lane iv, heating at 50 °C; lane v, heating at 75 °C; lane vi, heating at 100 °C. Right panel shows the relative intensity at different temperatures. The exposure times were the same in all procedures. Band intensities were analysed and compared using Image Lab software (Bio-Rad Laboratories) and GraphPad Prism version 6. ^**^Significantly different p < 0.01, ^***^p < 0.001, ^****^p < 0.0001. All values are means ± S.E. (error bars).

To optimise the fixation using organic solvent and heating, we varied the temperature and used different membranes (Fig. 1). Firstly, different fixation treatments on PVDF and nitrocellulose membranes were tested (Fig. 1a). Pooled human serum sample, containing 10 μg of protein, was resolved using 10% SDS-PAGE. Following this, the samples were used for Coomassie Brilliant Blue (CBB) staining (lane i) or electroblotted onto PVDF (Fig. 1a, top panel) or nitrocellulose membrane (Fig. 1a, bottom panel), then incubated with IgG antibody after the application of different fixative treatments, including no fixation (lane ii); drying at room temperature (lane iii); heating at 50 °C (lane iv); heating at 100 °C (lane v); immersion into the organic solvents (acetone and 50% methanol for PVDF and nitrocellulose membranes, respectively) (lane vi) at room temperature; immersion into the organic solvents at 0 °C (lane vii); immersion into the organic solvents at 0 °C followed by sample heating at 100 °C (lane viii) and immersion into the organic solvents at 0 °C followed by sample heating at 50 °C (lane ix). The results obtained using the PVDF membranes showed that every treatment has an effect on preventing protein loss from electroblotted membranes and increase its retaining compared to the traditional method, the difference in improving detection sensitivity between various fixation treatments and traditional method were analysed and showed in the right graph. These treatments lead to a higher intensity of the IgG signal, especially for that of the IgG light chain, a relatively low molecular weight protein. Furthermore, heating at 50 °C for 30 min (lane iv) was shown to improve IgG detection more than drying at room temperature (20–25 °C) (lane iii) or heating at 100 °C for 30 min (lane v). Acetone treatment at 0 °C (lane vii) led to better results than acetone treatment at room temperature for 30 min (lane vi), while the optimal condition of all was acetone treatment at 0 °C followed by heating at 50 °C (lane ix), both for 30 min. Furthermore, a large amount of protein was preserved on the membrane after fixation and we were able to detect degradation products of human IgG subclasses^17^ (asterisk, Fig. 1a, lane ix). These fragments were confirmed by analysing at least 30 μg of serum protein without the fixation step. To obtain a similar signal intensity to that of samples that underwent fixative treatment, 50 μg of serum protein was required for the analysis without applying the fixation step (Supplementary Fig. 1). We performed similar experiments using nitrocellulose membranes (Fig. 1a, bottom panel). Due to the different characteristics of nitrocellulose and PVDF membranes, different fixation procedures are required. Since nitrocellulose membranes dissolve in methanol, acetone, and other organic solvents^18^, we tested a series of acetone or methanol solutions, using water as the solvent. Of all the investigated fixatives, the application of 75% methanol, 50% acetone, and sample heating at 50 °C improved protein fixation the most. However, these treatments resulted in high intensity background signals. Optimal fixation was achieved when the membranes were incubated with 50% methanol/water (v/v) solution at 0 °C, followed by heating at 50 °C, both for 30 min.

Additionally, we investigated the temperature-dependence of the PVDF fixation process (Fig. 1b), the treatments including no fixation group (lane ii); heating at 25 °C (lane iii); heating at 50 °C (lane iv); heating at 75 °C (lane v); heating at 100 °C (lane vi). Results show that retained proteins were increased in the all tested temperatures compared to the traditional method. Furthermore, results indicate that the signal intensity of IgG markedly increased at 50 °C compared to the intensity at 25 °C. However, the detection of the protein bands decreased gradually with the increase in temperature beyond 50 °C. This demonstrated that the optimal fixation temperature for detection using anti-IgG antibody is 50 °C.

### Effects of the fixation step on sensitivity of immunoblotting

**Figure 2:**
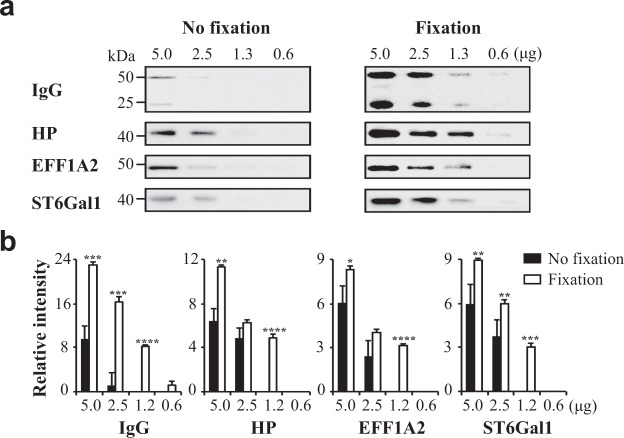
Effects of sample fixation on the retention of proteins of the method using PVDF membranes. (**a**) Indicated numerals are amounts (5.0, 2.5, 1.3, and 0.6 μg) of the pooled serum proteins were subjected to 10% SDS-PAGE. The blotted membranes were treated using the traditional (left panel) or optimised fixation protocol (right). (**b**) Staining intensities were statistically analyzed (n = 3 individual experiments). Solid bar, no fixation; White bar, optimised fixation protocol. The exposure times were the same in all procedures. Band intensities were analysed and compared using Image Lab software (Bio-Rad Laboratories) and GraphPad Prism version 6. ^*^Significantly different p < 0.05, ^**^p < 0.01, ^***^p < 0.001, ^****^p < 0.0001. All values are means ± S.E. (error bars).

The sensitivity of immunoblotting coupled with the optimised fixation method was determined. Serial dilutions of the pooled human serum were separated by 10% SDS-PAGE, and the proteins were transferred onto PVDF membranes. These membranes were stained with anti-IgG, anti-haptoglobin (HP), anti-α2,6-sialyltransferases (ST6Gal1), and anti-eukaryotic elongation factor 1 alpha 2 (EEF1A2) antibodies, without pre-treatment (Fig. 2a, left) or following the optimised fixation method (Fig. 2a, right). The results showed that without the fixation step, 5 μg of serum protein was required for the visualisation of HP and IgG, two highly abundant proteins, while with the fixation step, the required amount was 1.3 μg of proteins, showing approximately four-fold increase in protein detection. The intensity of the methodology for the detection of ST6Gal1 and EFF1A2, proteins with low abundance in sera, was shown to increase two-fold following sample fixation. The intensity analysis of the retention of proteins following the fixation were shown to increase 1.8- to 16-fold, compared with those in the samples without pre-treatment (Fig. 2b). Similar results were obtained using nitrocellulose membranes (Supplementary Fig. 2), where the intensity increased approximately 1.6- to 7.6-fold.

### Sample fixation prior to LB

**Figure 3:**
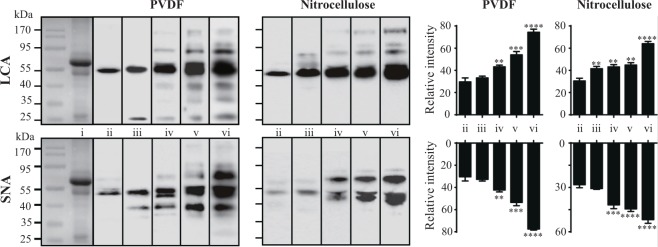
Fixation-dependent differences in lectin staining intensities when using PVDF and nitrocellulose membranes. Pooled human serum proteins (3 μg) were separated on 10% SDS-PAGE, and the proteins were transferred onto PVDF and nitrocellulose membranes, the whole membrane was cutted into five pieces for subsequently fixation treatments and followed by staining with lectins (LCA and SNA). Lane i, CBB staining; lane ii, no fixation; lane iii, drying at room temperature; lane iv, sample heating at 100 °C; lane v, organic solvent (acetone and 50% methanol for PVDF and nitrocellulose membranes, respectively) treatments at room temperature; lane vi, organic solvent treatments followed by sample heating at 100 °C. All treatments were applied for 30 min. Left, WB pattern; right, quantitative analysis (n = 3 individual experiments). The exposure times were the same in all procedures. Band intensities were analysed and compared using Image Lab software (Bio-Rad Laboratories) and GraphPad Prism version 6. ^**^Significantly different p < 0.01, ^***^p < 0.001, ^****^p < 0.0001. All values are means ± S.E. (error bars).

Pooled human serum protein samples (3 μg) were used for the optimization of the fixation step coupled with the lectin blotting (LB) procedure with *Lens culinaris* agglutinin (LCA) and *Sambucus nigra agglutinin* (SNA) lectins (Fig. 3). All applied fixation methods (Fig. 3, lanes iii-vi) allowed an effective prevention of protein removal from the membranes during LB, and the difference in improving detection sensitivity between various fixation treatments and traditional method were analysed and showed in the right graph. The optimal fixation method for PVDF membranes, was acetone treatment at room temperature followed by sample heating at 100 °C, both for 30 min. In the case of nitrocellulose membranes, the optimal fixation method was a combination of incubation in 50% methanol solution and heating at 100 °C for 30 min each. More protein bands were observed while using PVDF membranes, in both cases of procedures with and without fixation, compared with those observed when using nitrocellulose membranes. This discrepancy is due to the higher mobility of low molecular-weight proteins in the nitrocellulose membranes and decreased protein binding^19^.

### Sensitivity of LB

**Figure 4:**
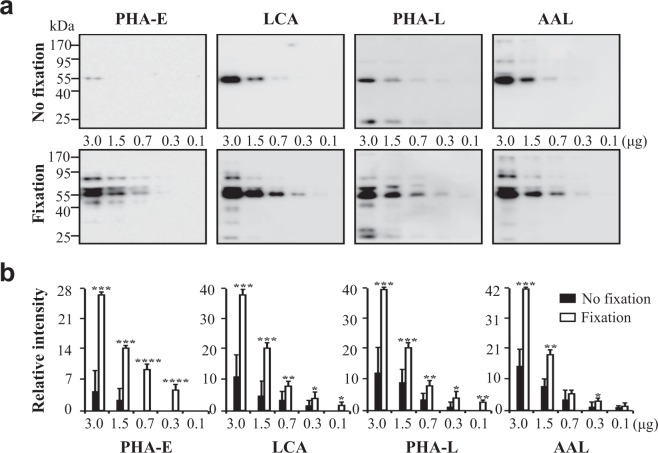
Sensitivity of the LB method coupled with the fixation step, when using PVDF membranes. Indicated numerals are amounts (3.0, 1.5, 0.7, 0.3, and 0.1 μg) of the pooled serum proteins were subjected to 10% SDS-PAGE. (**a**) The blotted membranes were treated using the traditional (top panel) or optimised fixation protocol (bottom panel). (**b**) Quantification of band intensities were statistically analyzed (n = 3 individual experiments). Solid bar, no fixation; White bar, sample fixation. The exposure times were the same for the same lectin blotting using fixation or no fixation. Band intensities were analysed and compared using Image Lab software (Bio-Rad Laboratories) and GraphPad Prism version 6. ^*^Significantly different p < 0.05, ^**^p < 0.01, ^***^p < 0.001, ^****^p < 0.0001. All values are means ± S.E. (error bars).

The sensitivity of the LB coupled with the identified optimal fixation method was further investigated. Serial dilutions of the pooled human serum proteins were separated by 10% SDS-PAGE, and the proteins were transferred onto PVDF membranes. The membranes were stained with lectins, *Phaseolus vulgaris* erythroagglutinin (PHA-E), LCA, *Phaseolus vulgaris* leucoagglutinin (PHA-L), and *Aleuria aurantia* lectin (AAL), combined with either no pre-treatment or with the identified optimal fixation method (Fig. 4). All types of lectins were shown to stain more glycoproteins when the analysis was combined with the fixation step (Fig. 4a, bottom panel) than without it (Fig. 4a, top panel), which was especially prominent for the glycoproteins with small molecular weight. These glycoproteins were not detected without prior fixation. Band intensities were analysed (Fig. 4b), and the sensitivity was shown to increase from 2.8- to approximately 15-fold when the method was coupled with the fixation step. Additionally, the ability of this fixation method in retention of protein when using nitrocellulose membranes, was determined, and shown to increase approximately from 3.7- to 12-fold (Supplementary Fig. 3).

### Applications of the optimised method in immunostaining and lectin staining

**Figure 5:**
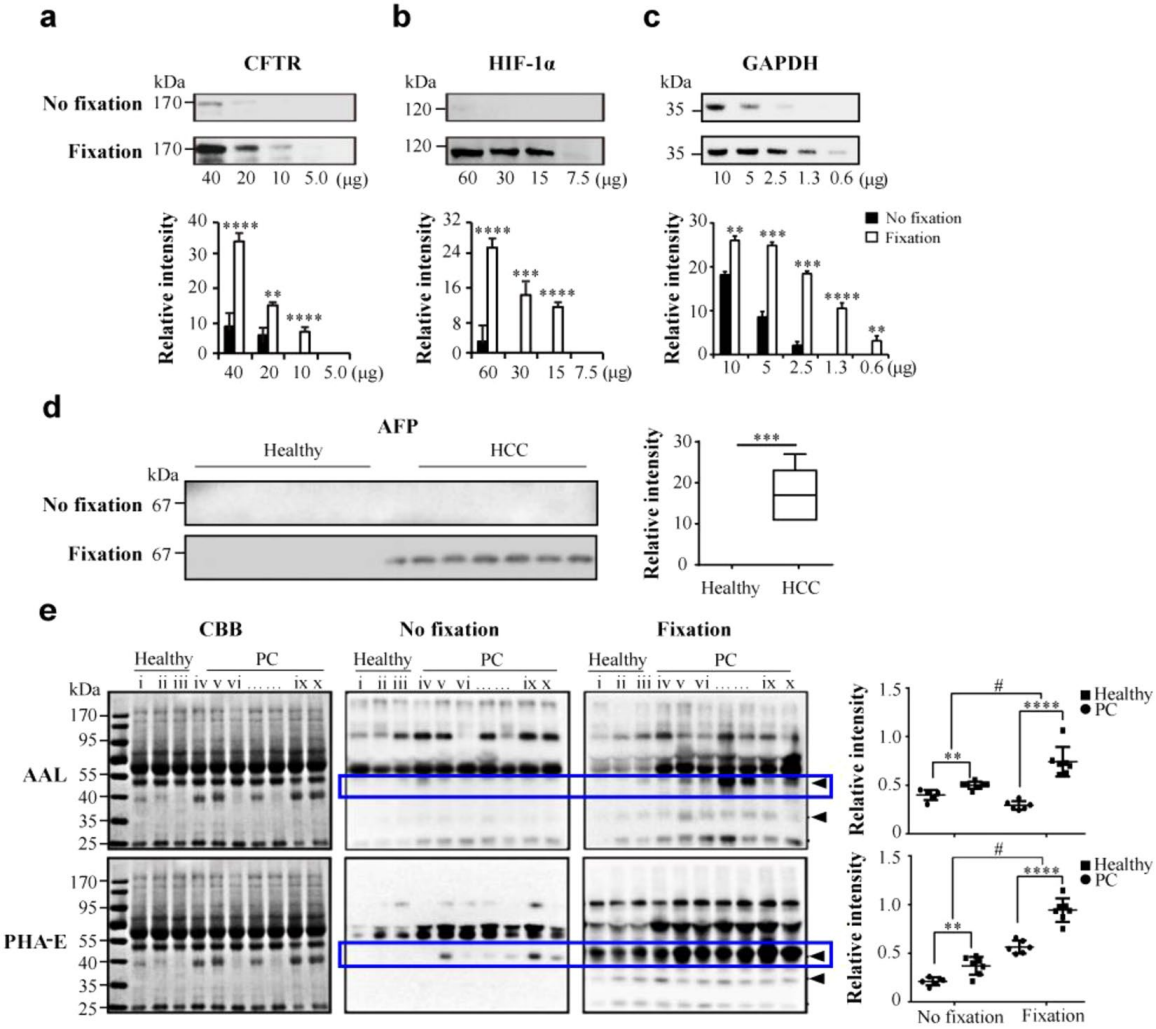
Application of the optimised immunostaining and lectin staining methods. (**a**) CFTR levels in HT-29 cells. (**b**) HIF-1α levels in HEK-293T cells. (**c**) GAPDH levels in liver tissue of mouse. Various amounts (quantity represented in μg) of total cellular proteins analysed using 8% SDS-PAGE and immunostained using PVDF membrane and treated with or without fixation treatments. (**d**) AFP levels in the sera of healthy volunteers (n = 6) and HCC patients (n = 7), with different sample volumes using the PVDF membranes, with or without the fixation. ^**^Significantly different p < 0.01, ^***^p < 0.001, ^****^p < 0.0001. All values are means ± S.E. (error bars). (**e**) AAL and PHA-E staining, using 6 μg of proteins from the sera of healthy volunteers (n = 6) and prostate cancer patients (PC, n = 10), blotted on PVDF membranes, with or without fixation. Three representative healthy samples (lane i, ii, iii) and seven representative prostate cancer samples (lane iv-x) are presented (left). Boxplot provides the quantification of the total band intensities (right). Circle, healthy subjects; square, prostate cancer patients. Student’s t-test. ^**^P < 0.01 and ^****^P < 0.0001, healthy subjects vs. PC patients; ^#^P < 0.0001, No fixation vs fixation groups. Band intensities were compared using Image Lab software (Bio-Rad Laboratories) and GraphPad Prism version 6.

Immunoblotting and LB are used for the determination of protein expression and glycan level variations across different populations or conditions. CFTR, expressed in many epithelial tissues, is a key membrane protein in the complex network of molecules involved in epithelial ion transporters regulating epithelial permeability^20^. HIF-1, a heterodimer composed of a constitutively expressed HIF-1β subunit and a hypoxic response factor HIF-1α subunit, activates the transcription of genes that are related to critical aspects of cancer biology^21^. AFP is the only serum marker currently approved for clinical use in HCC diagnostics^22^. We not only analysed the expression levels of CFTR in HT-29 cells, HIF-1α in HEK-293T cells, GAPDH in liver tissue of mouse, and serum AFP in HCC patients, but also the glycosylation changes in the sera of prostate cancer patients, in combination with the optimised fixation method. In our analysis of CFTR expression in HT-29 cells (Fig. 5a), two bands were detected on membranes when blotting was performed either with or without the fixation step, which is consistent with a previous study^23^. However, for the visualisation of CFTR, the amount of total cellular protein required in no fixation group was 40 μg (Fig. 5a, top panel), while 10 μg was enough for visualisation in the optimised fixation group (Fig. 5a, bottom panel). In HIF-1α staining (Fig. 5b), the band was barely detectable even with 60 μg of protein sample in the no fixation group (Fig. 5b, top panel); in contrast, signal intensity was shown to increase eight-fold following sample fixation (Fig. 5b, bottom panel). We also tested tissue protein using this optimal fixation (Fig. 5c). The results showed that without the fixation step, 2.5 μg o tissue protein was required for the visualisation of GAPDH, while with the fixation step, the required amount was 1.3 μg of proteins, showing approximately two-fold increase in protein detection. For immunostaining of AFP (Fig. 5d), six and seven serum samples respectively obtained from healthy subjects and HCC patients were used. The increased AFP levels in the sera of the HCC patients were not detected using conventional procedure with 10 μg of total serum proteins (Fig. 5d, top panel). On the other hand, using the same amount of total protein, AFP expression was detected following the application of the fixation step (Fig. 5d, bottom panel). The elevation of serum AFP in HCC observed in our study was consistent with a previous report^22^.

Additionally, AAL and PHA-E lectins were used to analyse altered glycosylation in prostate cancer (PC) patients (Fig. 5e). The levels of fucosylated proteins and glycoproteins with bisecting glycosylation were significantly increased in the sera of prostate cancer patients, in comparison with those in the healthy subjects (P < 0.01), in both no fixation and fixation groups. The results obtained using the AAL blotting are consistent with those previously described for prostate cancer patients^24^. Furthermore, in both the AAL and PHA-E staining, the application of fixation led to detection of a larger number of bands than that obtained without fixation, allowing a more precise analysis of the differences between healthy and prostate cancer patient samples. The fucosylated protein (43 kDa) (framed in blue box in AAL staining) was detected in the sera of prostate cancer patients only following application of the fixation step combined with staining for AAL. In the PHA-E staining, a 40-kDa protein (framed in blue box) was detected only in the sera of the prostate cancer patients when no fixation procedure was applied, while it was detected in the sera of both healthy subjects and prostate cancer patients following the application of the optimised fixation step.

## Discussion

In this study, we have developed an optimised method for the fixation of proteins on immunoblot membranes prior to the blocking step. This method is suitable for the detection of proteins from different sources, including cell lysates, tissue samples and sera. Our method was developed for application on both PVDF and nitrocellulose membranes during immunoblotting and LB, and shows significant advantages in the detection of low molecular weight proteins.

PVDF membrane is an extremely hydrophobic surface, binding proteins through the dipole and hydrophobic interactions^19^. The treatment of proteins with acetone is a common preparation method in proteomic research. Heating of the blotted membrane accelerates the evaporation of water, providing a more favourable environment for the binding of proteins to the PVDF membrane. Additionally, prolonged application of heat (30 min) leads to a decrease in the compactness of the denatured protein, which changes its structure from the persistent hydrophobic clustering and residual secondary forms to a loose structure, where the hydrophobic side chains and compacted glycans are exposed^25^. This denaturation allows the proteins to interact with the membrane through hydrophobic interactions, as well as the detection of glycans by using lectins. We showed that acetone treatment at 0 °C is more beneficial than that at room temperature, while heating of samples at 50 °C after the acetone treatment represents an additional improvement to this method. Taken together, our results indicate that the number of hydrophobic interactions increased during the moderate fixation step (heating at 50 °C and 75 °C); however, denaturation at higher temperatures leads to exposure of the internal hydrophobic groups and conformation changes, decreasing the accessibility of epitopes. Moreover, with the increase in protein denaturation, a decrease in epitope recognition by the specific antibodies was observed^23^. However, when fixing proteins during LB analysis, an increase in the degree of denaturation facilitated the detection of glycans.

Different nitrocellulose and PVDF membrane characteristics demand different fixation procedures. Due to the non-hydrophobic nature of the nitrocellulose membranes, proteins bind to these membranes through electrostatic interactions^26,27^, which results in lower protein-binding capacity for these membranes compared with that of the PVDF membranes (80 vs. 170 mg BSA bound/cm^2^)^19^. This was observed in our LB and immunoblotting experiments as well. Nitrocellulose membranes were readily dissolved in organic solvents such as acetone and methanol, and were shown to have a weak resistance to acidic and alkaline treatments^27^. Therefore, we explored several mixtures of water and different organic solvents. Among the conditions investigated, the application of 75% methanol, 50% acetone, and sample heating at 50 °C was shown to be more beneficial for protein fixation than the application of 50% methanol and heating at 50 °C. However, owing to the high background signal of the former method, the latter was selected as the optimal fixation method.

Immunostaining of proteins on cellulose acetate membranes usually involves acid fixation using sulfosalicylic acid or trichloroacetic acid^28,29^. Acid can hydrolyse sialic acid, an important terminal sugar of the glycoprotein carbohydrate chains. Protein fixation using our method did not remove sialic acid, as demonstrated by the analysis using SNA.

In order to verify the universality and significant advantages of these optimised methods, proteins from total cell lysates were particularly tested. CFTR protein was difficult to study and analyse due to the low levels and high turnover rates in the cells^30^. The optimised fixation method significantly facilitated its detection. HIF-1α protein was shown to be degraded under normoxic conditions and this made its detection difficult^31^. However, it was clearly detected even with very small amount of total protein following the application of the optimised method. Besides, HIF-1α protein levels increased in response to hypoxia^31^, which were also testified in our study (Supplementary Fig. 4). Furthermore, determination of *N*-glycan levels in the sera of prostate cancer patients using this optimised method helped us identify a fucosylated 43-kDa protein, which may represent a novel diagnostic marker. Additionally, as for the fucosylated 30-kDa and 25-kDa proteins and 40-kDa bisecting glycoprotein, they were initially detected only in the prostate cancer patients when no fixation step was used, which may suggest that these proteins are specific for the prostate cancer patients. However, the analysis coupled with the sample fixation step demonstrated that these proteins can be found in healthy subject sera as well, at lower levels. Here, we report the alterations in bisecting *N*-glycans in the sera of prostate cancer patients, which is catalysed by glycosyltransferase GnT-III. Previously, studies focused on the β1,6-branched oligosaccharides to explore potential relationships between prostate cancer progression and glycosyltransferase GnT-V or GnT-IX (Vb)^32,33^. Therefore, we demonstrated that this fixation method allows identification of proteins associated with the development of cancer and other diseases.

Other factors, such as the amount of SDS in the gel, buffer composition, transfer and blocking duration, blotting methods, and different ECL detection reagents, affect the immunoblot analysis as well. Here, we investigated the optimal method of retaining the separated proteins on the membranes after their transfer. Further studies should explore and define the optimal western blotting conditions through a comprehensive evaluation of a variety of factors. The increased sensitivity of the newly developed fixation step allows the detection of trace proteins and subtle differences between different samples, which may facilitate the identification and development of clinically useful biomarkers.

## Methods

### Lectins and antibodies

Biotinylated lectins, LCA, SNA, PHA-E, PHA-L, and AAL (Supplementary Table 1), were purchased from Vector Laboratories Inc. (Burlingame, CA, USA). Horseradish-peroxidase (HRP)-conjugated Affinipure Donkey anti-human IgG, rabbit anti-EFF1A2 and anti-GAPDH polyclonal antibodies were purchased from Proteintech Group (Chicago, IL, USA); rabbit anti-CFTR polyclonal antibody was from Alomone Labs (Jerusalem, Israel); rabbit anti-HIF1α polyclonal antibody was from Santa Cruz Biotechnology, Inc. (Santa Cruz, CA); rabbit anti-HP polyclonal antibody and rabbit anti-AFP polyclonal antibody were purchased from Boster (Wuhan, China); rabbit anti-mouse α2,6-sialyltransferase Affinity-Purify antibody was obtained from Immuno Biological Laboratories Co, Ltd. (Japan). Horseradish peroxidase-labelled anti-rabbit IgG antibody was obtained from Beyotime (Shanghai, China), deferoxamine mesylate salt (DFO) was purchased from Sigma (St. Louis, MO, USA).

### Serum samples

Serum samples were collected from prostate cancer (n = 10), HCC patients (n = 7), and healthy volunteers (n = 6) at the Dalian Municipal Central Hospital affiliated to the Dalian Medical University, China. The clinical characteristics of these patients are presented in Table 1. For patient with PSA levels > 4.5 μg/L or AFP levels > 1000 μg/L were selected as prostate cancer and HCC cases, respectively. All patients were diagnosed by pathological examination, and the specimens were obtained prior to surgery or biopsy. The specimens were not treated with protease inhibitors, and stored at −80 °C until further analyses. Each aliquot had been thawed no more than two times before use. For the preliminary studies, serum samples from six healthy volunteers were analysed. These specimens were stored at −80 °C in the specimen bank for less than 6 months. Informed consents were obtained from all patients in this study and all research protocols were approved by the Institutional Review Board of Dalian Medical University, China, in accordance with the established guidelines for the use of patients’ information and samples.

**Table 1:** Summary of Patient Information.

Group	n	age (y)	PSA level	AFP level
Healthy subject	6	46 ± 8	N/A	N/A
Prostate cancer	10	56 ± 7	5.5 (4.5–8.1)	N/A
Hepatocellular carcinoma	7	53 ± 8	N/A	>1000

N/A, not applicable. Age, mean value ± SD. PSA levels (ng/mL) are presented as median values.

### Cell culture

Human colon carcinoma HT-29 cells and human HEK-293T cells were acquired from the American Type Culture Collection (Manassas, VA, USA). HT-29 cells were cultured in 1640 medium (Sigma). HEK-293T cells were cultured in Dulbecco’s Modified Eagle Medium (DMEM, GIBCO). Both media were supplemented with 10% foetal bovine serum (FBS, GIBCO), 2 mM L-glutamine, 100 units/mL penicillin, 100 μg/mL streptomycin in a 37 °C incubator with 5% CO_2_ and 95% humidity. Chemical hypoxia was generated by treating HEK-293T cell with 150 μM DFO for 24 h and 48 h. Cells were then lysed and analysed for immunoblotting.

### Tissue protein

Liver tissue of ICR mice was snap-frozen in liquid nitrogen and ground; the powder was homogenized in 1 mL of lysis buffer containing a protease inhibitor mixture and centrifuged. All animal experiments were approved by the Animal Care and Use Committee of Dalian Medical University.

### Gel electrophoresis

Total protein concentrations were determined using the BCA protein assay kit (Takara Bio Inc.). Serum proteins and cellular proteins were incubated with SDS-PAGE sample buffer (125 mM Tris-HCl, pH 6.8, 4% SDS, 20% glycerol, 10% beta-mercaptoethanol, and 0.004% bromophenol blue) at 100 °C for 5 min. Tris-glycine SDS running buffer (25 mM Tris, 250 mM glycine, 0.1% SDS) was used for electrophoresis. After electrophoresis, proteins were transferred onto the Immobilon-P PVDF membrane or nitrocellulose membranes (Millipore) at 80 mA for 1 h by using Tris-glycine SDS (48 mM Tris, 39 mM glycine, 0.037% SDS) transfer buffer with 20% methanol and then stained with Coomassie Brilliant Blue (CBB) R-250 dye for the rapid reversible detection of protein bands for western blotting or LB analyses.

### Conventional western blotting

For the conventional western blotting, the blotted PVDF membranes were directly blocked with a blocking buffer (5% bovine serum albumin (BSA) in Tris-buffered saline (TBS) with 0.05% Tween-20 (TBST)) for 1 h and then probed with primary antibodies or lectins. After washing with TBST 3 times, the immunoreactive bands were detected by ChemiDoc XRS Image System (Bio-Rad Laboratories, Hercules, CA, USA). Quantification of protein bands was performed using Image Lab software.

### Fixation for immunoblotting

For immunoblotting, the electroblotted PVDF membranes were immersed in acetone at 0 °C for 30 min with gentle shaking, which was followed by heating at 50 °C for 30 min. The fixed membranes were briefly immersed in methanol and then in TBST for several minutes before their blocking with 5% BSA in TBST for 1 h. After washing with TBST (5 min, three times), the membranes were incubated with HRP-conjugated donkey anti-IgG (1:10,000), rabbit anti-EFF1A2 (1:5,000), anti-HP (1:400), anti-α2,6-sialyltransferase (1:200), anti-CFTR (1:500), anti-HIF-1α (1:500), and anti-GAPDH (1:3000), and anti-AFP (1:100) antibodies at 4 °C overnight. After washing with TBST (5 min, three times), the membranes were incubated with an enzyme-labelled goat anti-rabbit IgG antibody for 1 h at room temperature. The binding was visualized using the ECL (enhanced chemiluminescence) Plus reagents (Beyotime). When the nitrocellulose membranes were used, the organic solution was 50% methanol/water mixture applied at 0 °C. For immunostaining using the same antibody, the exposure times when both the traditional and the optimised fixation methods were applied, were the same.

### Fixation and LB

For LB, the fixation of the electroblotted membranes was performed by immersing the membranes in acetone at room temperature for 30 min, accompanied by gentle shaking, and followed by sample heating at 100 °C for 30 min. The subsequent procedure was the same as that described for the immunostaining. Finally, the membranes were incubated with biotinylated lectins (LCA, 1:15,000; SNA, 1:15,000; PHA-E, 1:20,000; PHA-L, 1:1,000; and AAL, 1:15,000; all diluted in TBST) at 4 °C overnight. After an additional washing with TBST (5 min, three times), the membranes were incubated with streptavidin-HRP diluted with TBST for 1 h at room temperature. For LB using the same lectin, the exposure times for samples by traditional method and the fixation method were the same.

### Statistical analysis

Band intensities were analysed and compared using Image Lab software (Bio-Rad Laboratories) and GraphPad Prism version 6. All experiments were performed at least three times. Data from western blotting or lectin blotting were analysed with Student’s t-test (two-tailed, unequal variance). The significance of differences was considered significant when P < 0.05.

## Supplementary information

Dataset 1

## Data Availability

All data used in this work are available upon request.
