# Supplementary material for: A fixation method for the optimisation of western blotting
Source: Sci Rep. 2019 Apr 30;9:6649. doi: 10.1038/s41598-019-43039-3 (PMC6491546; doi:10.1038/s41598-019-43039-3)
Supplement: Supplementary file 1 — Dataset 1 [file 41598_2019_43039_MOESM1_ESM.pdf]

A fixation method for the optimisation of western blotting

Jing Xu[1, #], Hebin Sun[1, #], Guoling Huang[1], Gang Liu[1], Zhi Li[2], Hui Yang[1], Lingling Jin[1], Xiaolin Cui[1], Lei Shi[1], Tonghui Ma[1], Akihiko Kameyama[3] and Weijie Dong[1*]

[1] College of Basic Medical Sciences, Dalian Medical University, Dalian 116044, Liaoning, China

[2] Clinical Laboratory, Dalian Municipal Central Hospital, 826-Xinan Road, Shahekou District, Dalian city, Liaoning 116033, China

[3] Biotechnology Research Institute for Drug Discovery, National Institute of Advanced Industrial Science and Technology (AIST), Open Space Laboratory C-2, 1-1-1 Umezono, Tsukuba, Ibaraki 305-8568, Japan

[*]Corresponding author information:

Weijie Dong, College of Basic Medical Sciences, Dalian Medical University, 9-Western Section, Lvshun South Road, Liaoning 116044, China

Tel.: +86-411-8611-0313

E-mail address: wjdong@dlmedu.edu.cn

Supplementary data summary:

Supplement table 1. Lectins and their primary sugar specificity. Page 3.

Supplement figure 1. IgG immunostaining using the PVDF membranes, with and without prior protein fixation. Page 4.

Supplement figure 2. Effects of the sample fixation on the sensitivity of the method, using nitrocellulose membranes. Page 5.

Supplement figure 3. Sensitivity of the LB method coupled with the fixation step, when using nitrocellulose membranes. Page 6.

Supplement figure 4. Western blot analysis of HIF-1α protein by fixation method using PVDF membrane. Page 7.

Supplement figure 5. AAL and PHA-E staining of serum protein from prostate cancer patients. Page 8.

Supplement figure 6. Lower exposure images of Figure 1a. Page 9.

Supplement figure 7. Full blots of Figure 2. Page 10.

Supplement figure 8. Full blots of Supplementary Figure 2. Page 11.

Supplement figure 9. Full blots of Figure 5 a-d. Page 12.

Supplement figure 10. Western blot analysis of CFTR protein with and without fixation method using PVDF membrane, lower exposure images. Page 13.

Supplementary Information

Supplementary Table 1. Lectins and their primary sugar specificity.

Lectin	Common abbreviation	Primary sugar specificity
Lens culinaris lectin	LCA	Fucα1-6GlcNAc, α-Man, α-Glc
Sambucus nigra lectin	SNA	Sia2-6Gal/GalNAc
Phaseolus vulgaris erythroagglutinin	PHA-E	NA2 and bisecting GlcNAc
Phaseolus vulgaris leucoagglutinin	PHA-L	Tri- and tetra-antennary complex oligosaccharides
Aleuria aurantia lectin	AAL	Broad specificity to fucosylated glycans

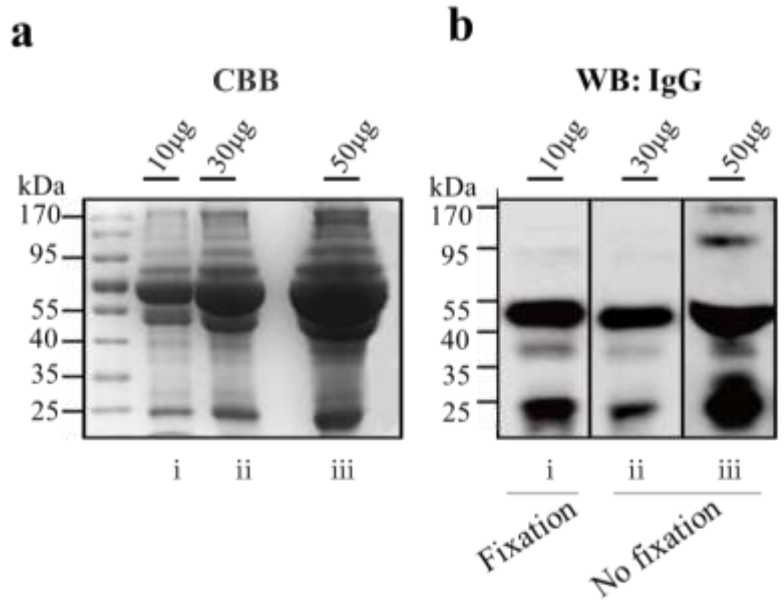

Supplementary Figure 1. IgG immunostaining using the PVDF membranes, with and without prior protein fixation. Different amounts of pooled human serum proteins were separated by 10% SDS-PAGE. (a) CBB staining. (b) IgG immunostaining, lane i, acetone treatment at 0 ℃ followed by the heating at 50 ℃, both for 30 min,10 μg of total proteins. lane ii, no fixation, 30 μg of total proteins; lane iii, no fixation, 50 μg of total proteins. The exposure time for samples by traditional method and the fixation method are the same.

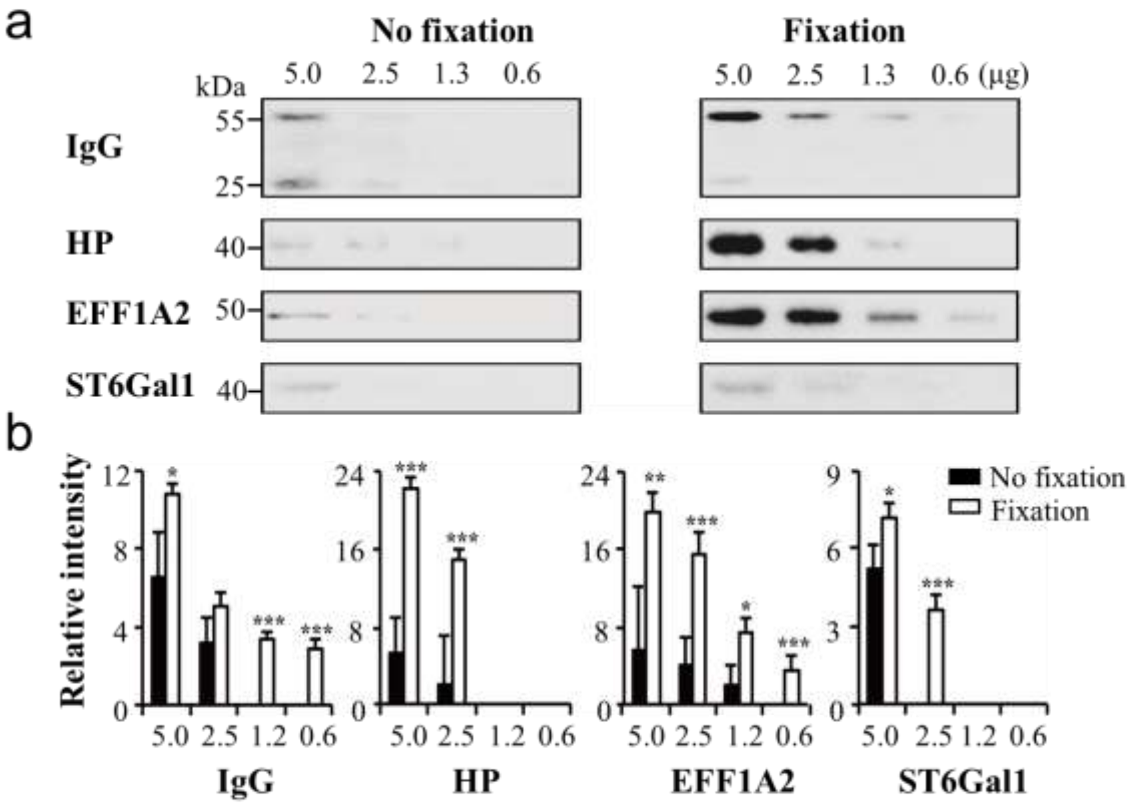

Supplementary Figure 2. Effects of the sample fixation on the sensitivity of the method, using nitrocellulose membranes. (a) Indicated numerals are amounts (5.0, 2.5, 1.3 and 0.6 μg) of the pooled serum proteins were subjected to 10 % SDS-PAGE. The blotted membranes were treated using the traditional (left panel) or optimised fixation protocol (right). (b) Staining intensities were statistically analyzed (n = 3 individual experiments). Solid bar, no fixation; White bar, optimised fixation protocol. The exposure times were the same in all procedures. Band intensities were compared using Image Lab software (Bio-Rad Laboratories) and GraphPad Prism version 6. *, significantly different $p < 0.05$, **, $p < 0.01$, ***, $p < 0.001$. All values are means ± S.E. (error bars).

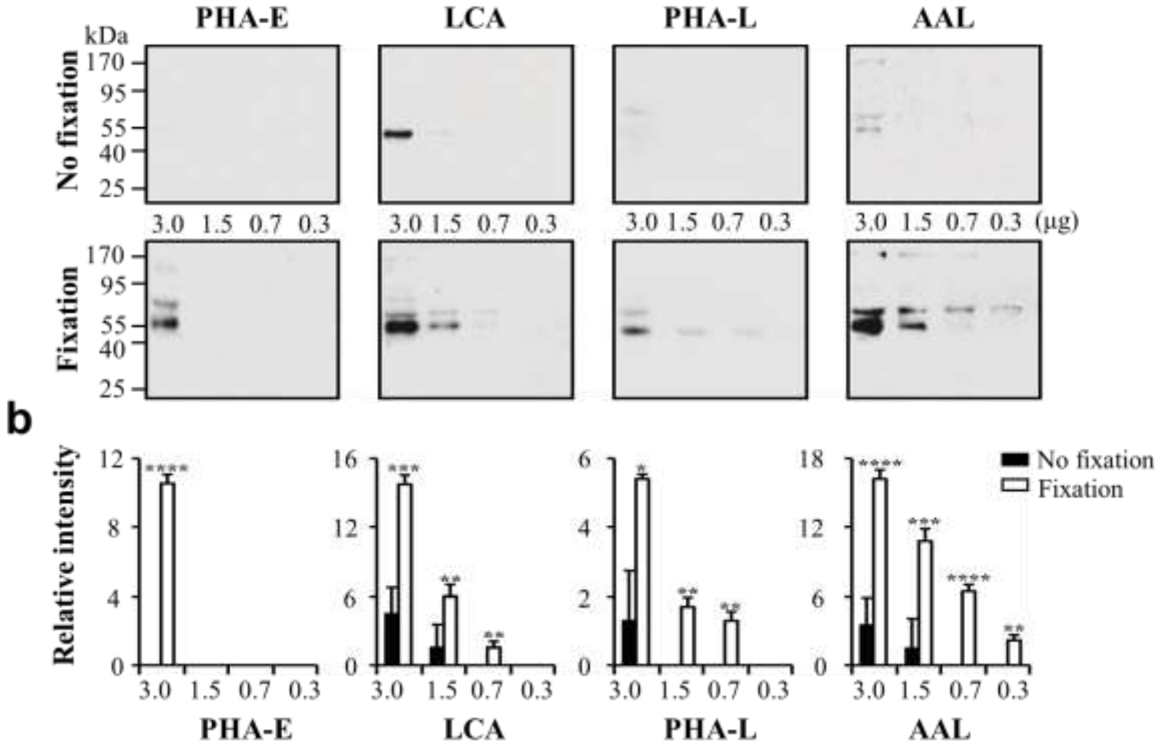

Supplementary Figure 3. Sensitivity of the LB method coupled with the fixation step, when using nitrocellulose membranes. Indicated numerals are amounts (3.0, 1.5, 0.7, 0.3, and 0.1 μg) of the pooled serum proteins were subjected to 10 % SDS-PAGE. (a) The blotted membranes were treated using the traditional (up panel) or optimised fixation protocol (down panel). (b) Quantification of band intensities were statistically analyzed (n = 3 individual experiments). Solid bar, no fixation; White bar, sample fixation. The exposure times were the same between different methods. Band intensities were compared using Image Lab software (Bio-Rad Laboratories) and GraphPad Prism version 6. **, significantly different p < 0.01, ***, p < 0.001, ****, p < 0.0001. All values are means ± S.E. (error bars).

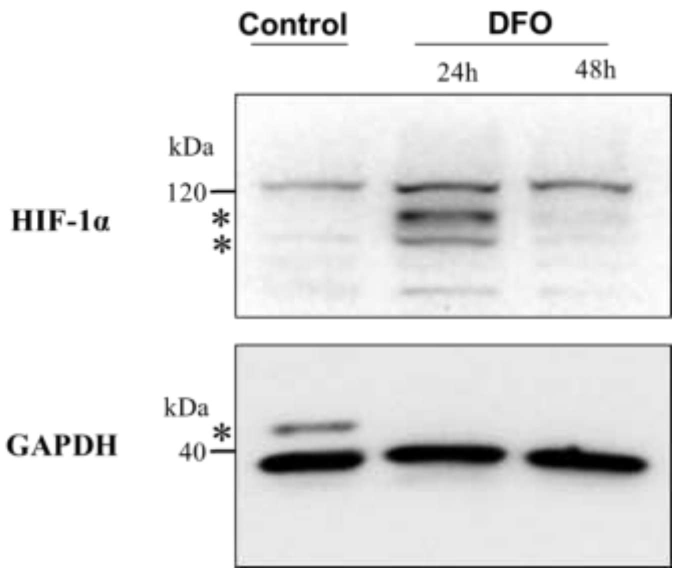

Supplementary Figure 4. Western blot analysis of HIF-1α protein by fixation method using PVDF membrane. Human HEK-293T cells were incubated under normoxia and the treatment with DFO (150 μM, 24 and 48-hour) to mimic hypoxia. 7.5 μg total cellular protein from above three different cells were analysed by anti-HIF-1α antibody after fixation treatment. GAPDH was used for an internal standard. The asterisks indicate nonspecific staining of antibodies.

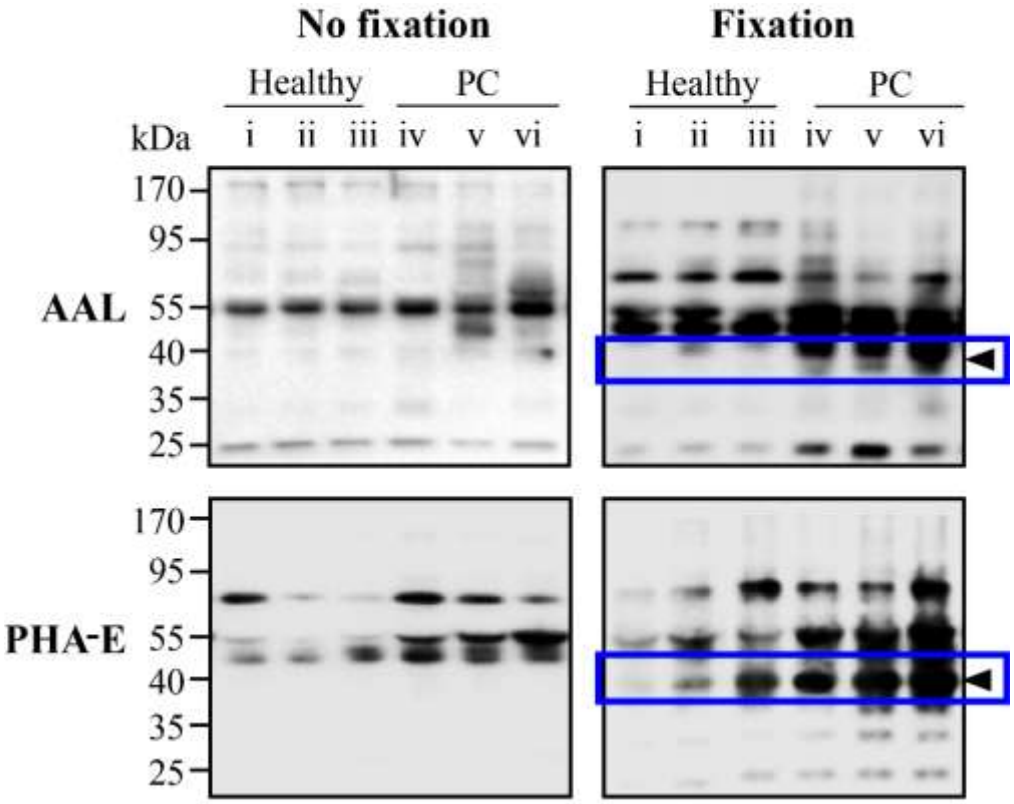

Supplementary Figure 5. AAL and PHA-E staining of serum protein from prostate cancer patients. 6 μg of proteins from the sera of healthy volunteers (n = 3) and prostate cancer patients (PC, n = 3), blotted on PVDF membranes, with or without fixation. Three representative healthy samples (lane i, ii, iii) and three representative prostate cancer samples (lane iv-vi) are presented.

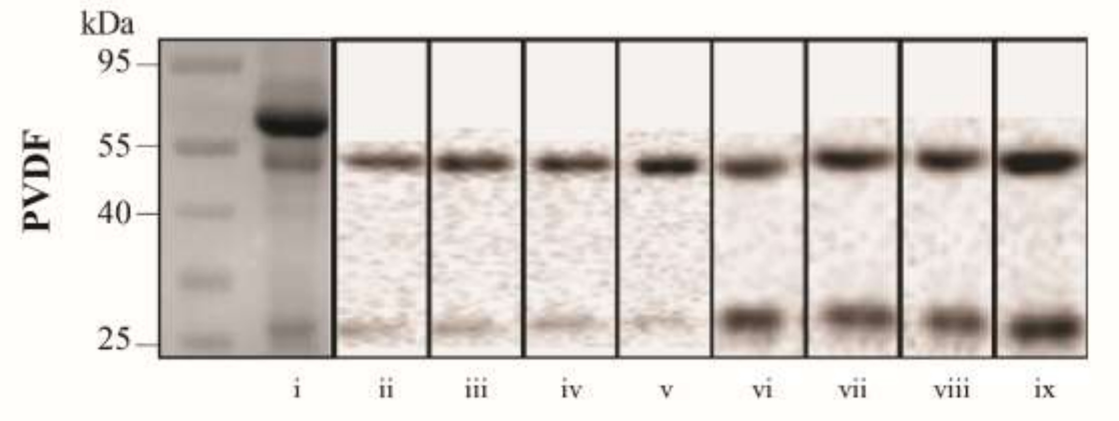

Supplementary Figure 6. Fixation method-dependent differences in the immunostaining intensity. Pooled human serum samples (10 μg) were analysed by 10 % SDS-PAGE, and the separated proteins were transferred onto PVDF membranes. Representative images, showing anti-human IgG antibody staining after the following treatments: lane i, Coomassie Brilliant Blue (CBB) staining; lane ii, no fixation; lane iii, drying at room temperature; lane iv, heating at 50 ℃; lane v, heating at 100 ℃; lane vi, immersion into the organic solvents (acetone and 50 % methanol for PVDF and nitrocellulose membranes, respectively) at room temperature; lane vii, immersion into the organic solvents at 0 ℃; lane viii, immersion into the organic solvents at 0 ℃ followed by sample heating at 100 ℃; lane ix, immersion into the organic solvents at 0 ℃ followed by sample heating at 50 ℃. All treatments were performed for 30 min.

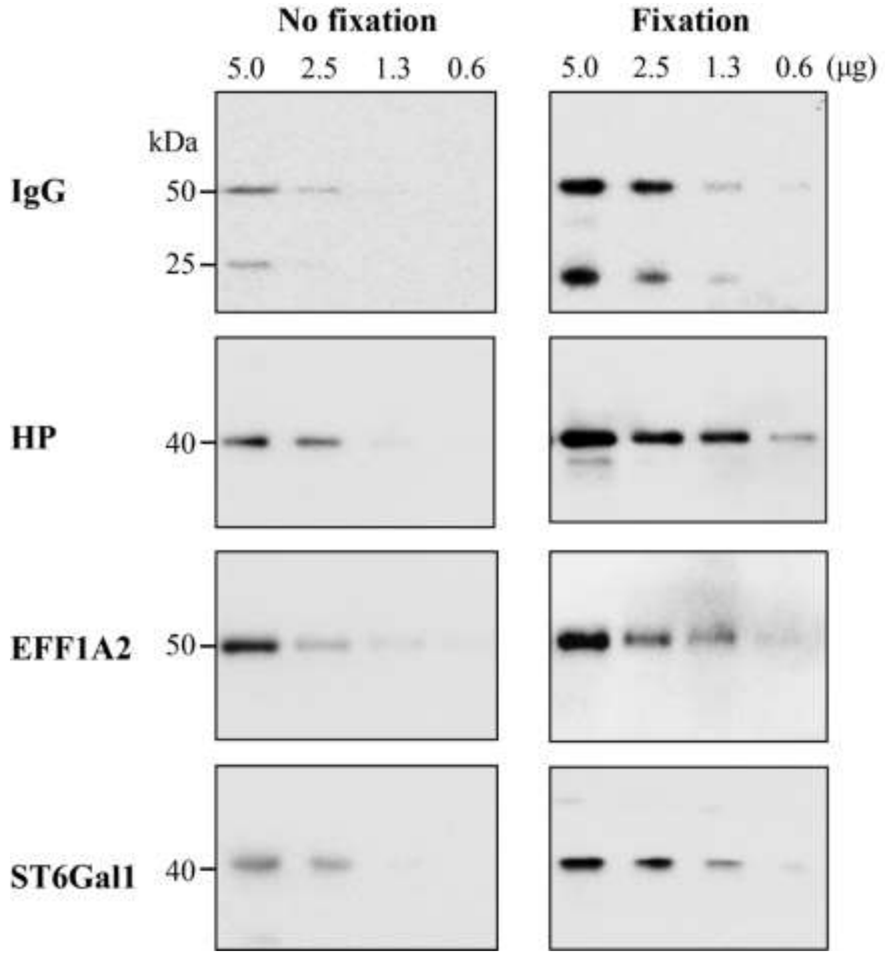

Supplementary Figure 7. Effects of sample fixation on the retention of proteins of the method using PVDF membranes. (a) Indicated numerals are amounts (5.0, 2.5, 1.3, and 0.6 μg) of the pooled serum proteins were subjected to 10% SDS-PAGE. The blotted membranes were treated using the traditional (left panel) or optimised fixation protocol.

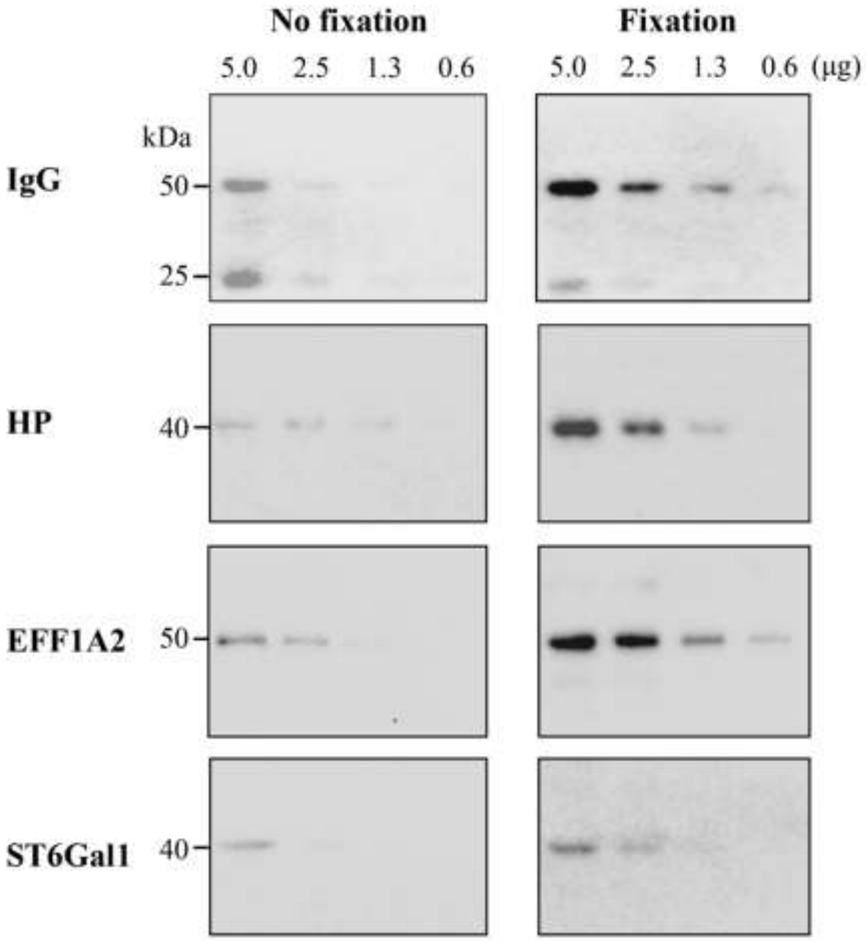

Supplementary Figure 8. Effects of the sample fixation on the sensitivity of the method, using nitrocellulose membranes. (a) Indicated numerals are amounts (5.0, 2.5, 1.3 and 0.6 μg) of the pooled serum proteins were subjected to 10 % SDS-PAGE. The blotted membranes were treated using the traditional (left panel) or optimised fixation protocol.

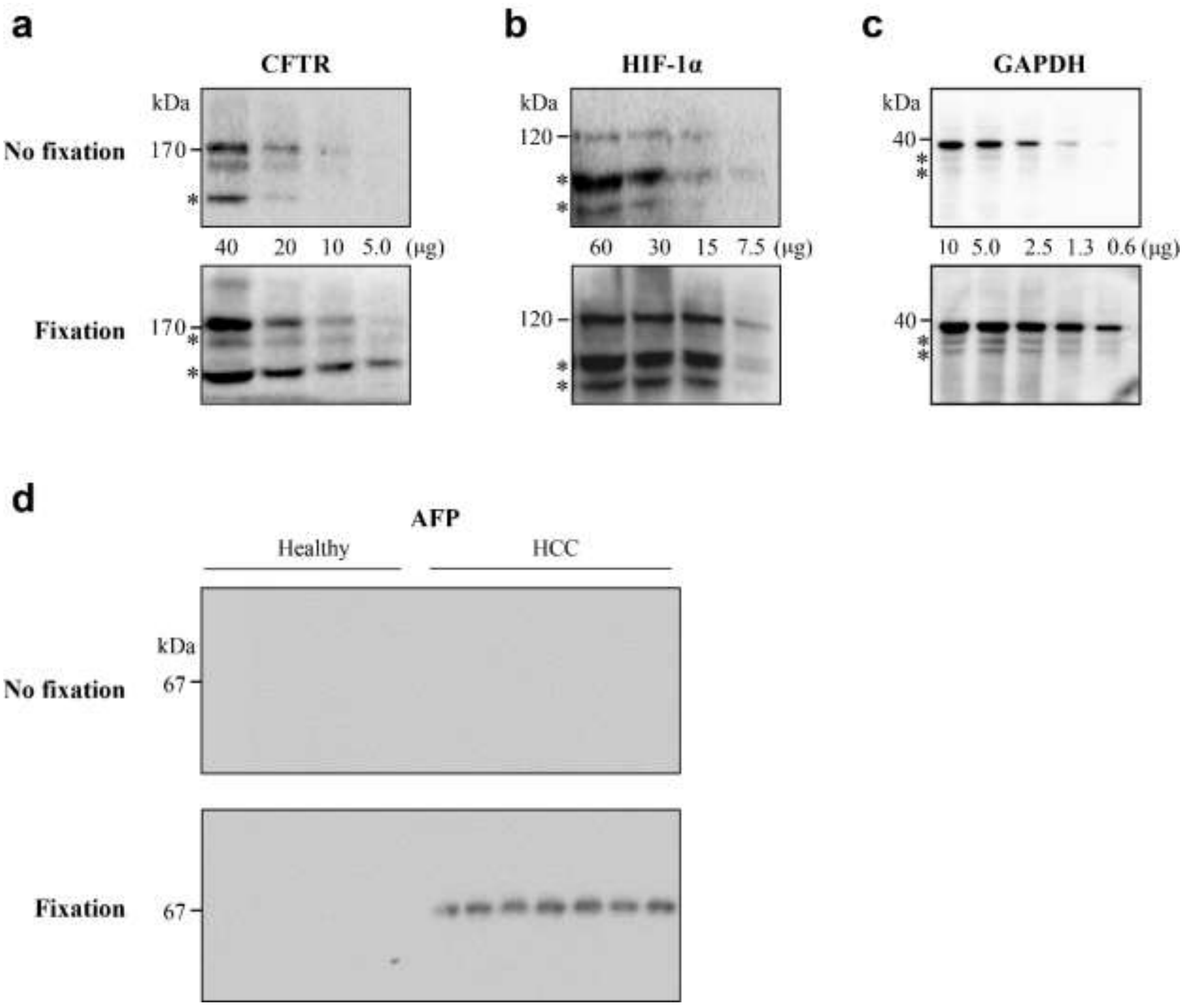

Supplementary Figure 9. Application of the optimised immunostaining and lectin staining methods. (a) CFTR levels in HT-29 cells. (b) HIF-1α levels in HEK-293T cells. (c) GAPDH levels in liver tissue of mouse. Various amounts (quantity represented in μg) of total cellular proteins analysed using 8 % SDS-PAGE and immunostained using PVDF membrane and treated with or without fixation treatments. (d) AFP levels in the sera of healthy volunteers (n = 6) and HCC patients (n = 7), with different sample volumes using the PVDF membranes, with or without the fixation. The asterisks indicate nonspecific staining of antibodies.

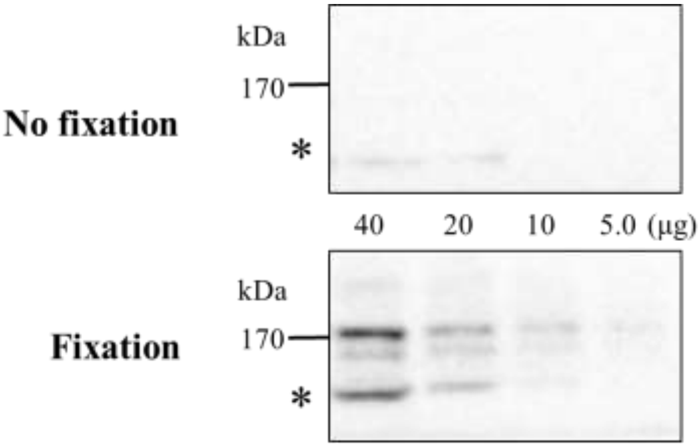

Supplementary Figure 10. Western blot analysis of CFTR in HT-29 cells. Various amounts (quantity represented in μg) of total cellular proteins analysed using 8 % SDS-PAGE and immunostained with CFTR using PVDF membrane and treated with or without fixation treatments. The asterisks indicate nonspecific staining of antibodies.
